# Widespread Striatal Delivery of GDNF from Encapsulated Cells Prevents the Anatomical and Functional Consequences of Excitotoxicity

**DOI:** 10.1155/2019/6286197

**Published:** 2019-03-11

**Authors:** Dwaine F. Emerich, Jeffrey H. Kordower, Yaping Chu, Chris Thanos, Briannan Bintz, Giovanna Paolone, Lars U. Wahlberg

**Affiliations:** ^1^Gloriana Therapeutics, Providence, Rhode Island, USA; ^2^Department of Neurological Sciences, Rush University Medical Center, Chicago Illinois, USA; ^3^Cytosolv, Providence, Rhode Island, USA; ^4^Department of Diagnostic and Public Health, Section of Pharmacology, University of Verona P.le, LA Scuro, Verona, Italy

## Abstract

**Methods:**

Human ARPE-19 cells engineered to secrete high levels of the glial cell line-derived neurotrophic factor (GDNF) were encapsulated into hollow fiber membranes. The devices were implanted into the rat striatum 1 week prior to striatal quinolinic acid injections. Animals were evaluated using a battery of validated motor tests, and histology was performed to determine the extent of GDNF diffusion and associated prevention of neuronal cell loss and behavioral deficits.

**Results:**

Encapsulated cell-based delivery of GDNF produced widespread distribution of GDNF throughout the entire implanted striatum. Stereological estimates of striatal neuron number and volume of lesion size revealed that GDNF delivery resulted in near complete neuroprotection.

**Conclusions:**

Delivery of neurotrophic molecules such as GDNF using encapsulated cells has reached a technological point where clinical evaluation is justified. Because GDNF has been effective in animal models of Parkinson's disease, stroke, epilepsy, and Huntington's disease, among other debilitating neurodegenerative diseases, encapsulated cell-based delivery of GDNF might represent one innovative means of slowing the neural degeneration seen in a myriad of currently untreatable neurological diseases.

## 1. Introduction

Treating neurodegenerative diseases is an urgent challenge. Neurotrophic factors are attractive therapeutic candidates because they can enhance neuronal functioning, are neuroprotective, and have the potential to reverse ongoing neurodegeneration that causes neurological deficits. While neurotrophic factors have been consistently effective in animal models [1–8], clinical development and evaluation has been limited. A major reason for the delayed development of effective neurotrophic therapies has been the inability to deliver them across the blood-brain barrier (BBB) directly to target sites in a stable, controlled, and continuous manner [9–13]. Several strategies are under development to optimize the diffusion and spread of trophic factors into the brain tissue. These include direct brain infusion [14], various gene therapy approaches [7, 8], cell therapies [15], and biomaterial-based drug-delivery systems [15]. Each approach has its own advantages and limitations, but none have yet produced significant enough efficacy to justify widespread clinical evaluation.

Here, we describe a novel means of delivering very high concentrations of neurotrophic factors directly to the site of neuronal damage using an encapsulated cell therapy technology [16, 17]. Cells are enclosed in a semipermeable capsule, which is then implanted into the brain. The capsule membrane allows oxygen and nutrients to enter and nourish the encapsulated cells while also allowing the therapeutic molecule of interest to leave the capsule and diffuse into the surrounding brain tissue. Immunological reactions to the encapsulated cells are reduced because the semipermeable membrane prevents elements of the host immune system from gaining access to the cells, thereby protecting against rejection. Indeed, even under xenograft conditions, the cells within the capsule remain viable without the need for immunosuppression. Furthermore, using human cells as the delivery vehicle further reduces the chances of immunological reactions. The cell line was produced using a transposon-based gene expression system resulting in high protein secretion 1-2 orders of magnitude higher than that used in previous cell encapsulation studies [18–20]. The cells were encapsulated within devices containing an optimized cell scaffolding previously shown to promote long-term cell viability in both animal models [21, 22] and recent human clinical trials in Alzheimer's patients [23]. In this study, we tested the hypothesis that encapsulated cell-based delivery of the neurotrophic molecule glial cell line-derived neurotrophic factor (GDNF) could result in widespread, but targeted, delivery of biologically active GDNF to the striatum. GDNF-secreting devices were implanted into the rodent striatum prior to quinolinic acid (QA) lesions. Comprehensive histological analysis and neurological testing revealed that GDNF was distributed throughout the striatum to exert a potent, essentially complete, neuroprotective effect. Together with previous demonstrations of long-term, controlled, safe, and targeted delivery of GDNF in small and large animal models, these data provide ongoing support for continued clinical development of this approach.

## 2. Methods and Materials

### 2.1. Subjects

Adult male Sprague-Dawley rats (Harlan Laboratories), ∼3 months old and weighing 225-250 grams, were housed in groups of 4 in a temperature- and humidity-controlled colony room maintained on a 12-hour light/dark cycle. Food and water were available *ad libitum* throughout the experiment. All experimentation was conducted in accord with National Institutes of Health guidelines.

### 2.2. Cell Culture

ARPE-19 cells were cultured using standard plating and passaging procedures in T-175 flasks with growth medium; DMEM+glutamax (1x) was supplemented with 10% fetal bovine serum (Gibco). Routine culture consisted of feeding the cells every 2-3 days and passaging them at 70-75% confluence. Cells were incubated at 37°C, 90% humidity, and 5% CO_2_.

### 2.3. Cell Line Establishment

Human GDNF cDNA optimized for human cell line expression was produced by Invitrogen, Denmark, and subsequently cloned to replace NGF in the expression vector pT2.CAn.hNGF [21], resulting in the plasmid pT2.CAn.hoG. ARPE-19 cells were transfected with this vector using the Sleeping Beauty (SB) transposon system as previously described [21, 22]. Briefly, cells were cotransfected with the plasmid pT2.CAn.hoG and the SB vector pCMV-SB-100x. As the SB vector does not contain a eukaryotic selection marker cassette, it is only transiently expressed. The transient expression window allows for the active, transposase-mediated integration of the SB transposon, i.e., the inverted repeat SB substrate sequences and the sequences contained within these repeats, including the GDNF expression and neomycin antibiotic resistance cassettes. Clones were selected using G418 (Sigma-Aldrich, Copenhagen, Denmark), and single colonies were expanded and isolated based on their GDNF release levels.

### 2.4. Device Fabrication

Cells were encapsulated into hollow fiber membranes manufactured from 7 mm segments of polyethersulfone membrane (Akzo, Germany) internally fitted with filaments of polyethylene terephthalate yarn scaffolding for cell adhesion. Prior to filling, cultured cells were dissociated and suspended in HE-SFM at a density of 8,333 cells/*μ*l. 6 *μ*l of cell solution (5 × 10^4^ cells in total) was injected into each device using a custom-manufactured automated cell-loading system. Devices were kept in HE-SFM at 37°C and 5% CO_2_ for either 3 weeks (low-dose group) or 12 weeks (high-dose group) prior to surgical implantation.

Previous studies [24] used scanning electron microscopy (SEM) to examine the morphology of these membranes. SEM cross sections of the polyethersulfone membrane confirmed that the membrane possessed a typical isoreticulated morphology with a relatively dense, thin outer skin and an open, much thicker macroporous substructure. Measures of membrane cross sections revealed an inner diameter of 481 *μ*m, an outer diameter of 663 *μ*m, and a corresponding wall thickness of approximately 90 *μ*m.

### 2.5. Surgery

Rats were anesthetized with isoflurane (3-4%) and placed into a stereotaxic instrument (Stoelting Inc.). A midline incision was made in the scalp, and a hole drilled for the unilateral placement of a device (7 mm in length) into the striatum using a stainless-steel cannula mounted to the stereotaxic frame. The coordinates for implantation with respect to the Bregma were as follows: AP: 0.0, ML: 3.2, and DV: -7.5. After placement of the device, the cannula was withdrawn and the skin sutured closed.

One week following device implantation, the rats were anesthetized with isoflurane and positioned in a stereotaxic frame for the injection of the QA (225 nmol). A 28-gauge Hamilton syringe was connected to the stereotaxic frame and lowered into the previously implanted striatum at the following coordinates with respect to the Bregma: AP: 1.0, ML: 2.6, and DV: -5.0. The QA was infused in a volume of 1 *μ*l per site over 5 minutes. The needle was left in place for an additional two minutes to allow the QA to diffuse from the injection site then removed and the skin sutured closed. The treated rats were allotted to 3 experimental groups: QA lesion only (*n* = 8), QA+GDNF low-dose (*n* = 8), and QA+GDNF high-dose (*n* = 6).

### 2.6. Neurological Evaluation

Using a validated battery of tests [25], the rats were evaluated to provide a behavioral measure of the extent of the lesion as well as the magnitude of benefit provided by the GDNF implants. All tests were conducted 24 hours prior to device implantation (baseline), 24 hours prior to QA injection (prelesion), and again 2 and 4 weeks postlesion. All testing was performed in a dim light testing room and the individual tests included (in order of testing) the following.

#### 2.6.1. Cylinder Test of Spontaneous Forelimb Use

The rats were placed individually in an acrylic cylinder (20 cm in diameter and 40 cm in height), and left and right forepaw contacts with the wall of the cylinder were quantified. Twenty total forepaw contacts were required to complete each testing session.

#### 2.6.2. Spontaneous Forelimb Placing Use

The forelimb placing test assessed the rat's ability to make directed forelimb movements in response to sensory stimuli. The rats were held with their limbs hanging unsupported and the length of their body parallel to the edge of a table. They were then raised and their whiskers were stimulated by brushing each side against the edge of the table. In naïve rats, this elicits a same-side forelimb response by placing the paw on the top of the table. Each rat received 10 consecutive trials with each forelimb.

#### 2.6.3. Stepping Test

Rats were placed on a flat surface and their hind legs gently lifted by raising their tail upward leaving the forelimbs resting on the table surface. The animal was pulled steadily backward, 1 meter over 30 seconds, and the adjusting steps were recorded for each forepaw.

### 2.7. GDNF ELISA

GDNF secretion from cell-loaded devices was confirmed prior to implantation and again following retrieval from the brain. Immediately following retrieval, all devices were incubated at 37°C in HE-SFM. Media samples (4-hour incubation) were collected the next day to quantify GDNF release using a commercially available kit (DuoSet® for human GDNF; R&D Systems, Minneapolis, MN).

### 2.8. Immunohistochemistry

Rats were deeply anesthetized and transcardially perfused with 200 ml of 0.9% ice-cold saline. Following saline perfusion, the rats were decapitated and the devices were removed and processed for GDF secretion as described below. The brains were placed into Zamboni's fixative for 1 week and then transferred to 25% sucrose for 48 hours. Frozen, 40 *μ*m thick coronal sections throughout the striatum and substantia nigra were cut and saved. An immunoperoxidase labeling method was used to visualize the volume of GDNF distribution in the rat striatum and substantia nigra while NewN-immunoreactive neurons were assessed in the striatum only. Endogenous peroxidase was quenched by 20-minute incubation in 0.1 M sodium periodate, and background staining was blocked by 1-hour incubation in a solution containing either 2% bovine serum albumin or 5% normal horse serum. Tissue sections were immunostained for GDNF (R&D Systems, AF-212-NA; 1 : 500) and NeuN (Millipore, MAB377; 1 : 1000) overnight at room temperature. After 6 washes, the sections were sequentially incubated for 1 hour in biotinylated horse anti-goat IgG (Vector Laboratories; 1 : 200) for GDNF and horse anti-mouse IgG (Vector Laboratories; 1 : 200) for NeuN followed by the *Elite* avidin-biotin complex (Vector Laboratories; 1 : 500) for 75 minutes. The immunohistochemical reaction was completed with 0.05% 3,3′-diaminobenzidine, 0.005% H_2_O_2_, and 0.05 M nickel (II) sulfate. Sections were mounted on gelatin-coated slides, dehydrated through graded alcohol, cleared in xylene, and cover-slipped with Cytoseal (Richard-Allan Scientific, Kalamazoo, MI).

### 2.9. Quantification

Optical fractionator unbiased sampling was used to estimate the total number of NeuN-immunoreactive neurons within the striatum [26, 27]. In each rat, we evaluated equispaced sections throughout the striatum length from its most anterior extent (Bregma + 2.2 mm) to the caudal level of the optic chiasm (Bregma − 1.3 mm). The striatum was outlined through a 1.25x objective using the Stereo Investigator software (MicroBrightField, VT), and the total number of NeuN-immunopositive neurons within the striatum was calculated for each animal. The Cavalieri estimator [26–28] was used to assess the volume of the striatum, the extent of QA lesion, and the GDNF distribution. Serial coronal sections extending throughout the striatum were sampled as described above using a 100 × 100 *μ*m point grid with a 10x objective. The volume of the QA lesion was assessed by quantifying the extent of the absent area, whereas GDNF distribution was quantified by measuring the spreading of GDNF immunoreactivity, and the effects of QA injections and GDNF treatments on the striatal neural population were assessed by counting NeuN-immunostained neurons. Quantification of the relative optical density (OD) of striatal GDNF immunoreactivity was performed using an Olympus microscope coupled to a computer-assisted morphometry system (Scion Image 1.63; NIH), as described previously [27]. The GDNF immunostaining throughout the striatum was identified and manually outlined. The OD was then automatically measured by using the NIH image software. To account for differences in background staining intensity, background OD measurements in each section were taken from corpus callosum-lacking GDNF immunoreactive profiles and then subtracted from the OD of each GDNF-stained striatum to provide a final OD value.

## 3. Results

### 3.1. GDNF ELISA

GDNF secretion from cell-loaded devices was confirmed prior to implantation and again following retrieval from the brain. In all animals, the implanted devices were easily retrieved with no host tissue adhering to the capsule wall, they were removed intact, and there was no evidence that any capsule broke either in situ or during the retrieval procedure. All implants were located centrally within the striatum. Dorsally, the devices extended through the corpus callosum, the overlying cortex, and extended ventrally to approximately the level of the anterior commissure. Prior to implantation, device secretion corresponded to 163 ± 5.0 ng/24 hours and 1056 ± 194.0 ng/24 hours for the GDNF low- and GDNF high-dose groups, respectively. Following the 4-week implant, GDNF levels were elevated relative to preimplant values reaching secretion levels of 893.9 ± 205.6 ng/24 hours in the low-dose group and 2046.2 ± 381.0 ng/24 hours in the high-dose group.

### 3.2. Histology

**Figure 1:**
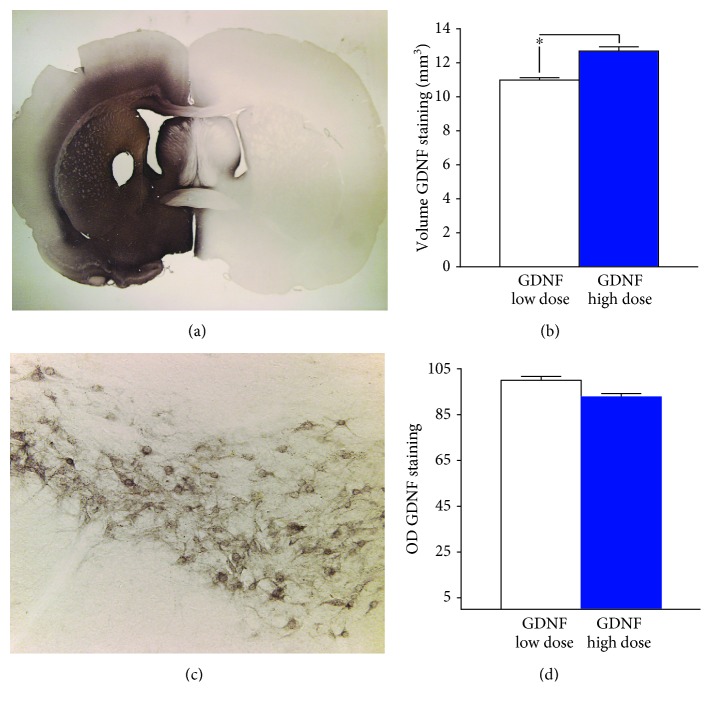
Widespread distribution of GDNF in the rat striatum. Photomicrographs of widespread GDNF immunoreactivity in the striatum of rats implanted with the lower dose GDNF-secreting device (a) and associated immunoreactivity in the substantia nigra (c). Histograms of the mean (±SEM) of the volume (b) and optical density (d) of GDNF staining. ^∗^*p* < 0.05.

Immunohistochemical staining of GDNF secreted from encapsulated cells revealed robust distribution of the growth factor in all animals. Intense GDNF immunoreactivity was seen throughout the striatum and into the globus pallidus and ventral pallidum. Immunoreactivity was also observed in the corpus callosum and the overlying cortex adjacent to the implant site (Figure 1(a)). Neurons within the pars compacta of the substantia nigra stained positive for GDNF, consistent with the retrograde transport of the protein (Figure 1(c)). The volume of striatal GDNF was 10.99 ± 1.1 mm^3^ for the low-dose group and 12.69 ± 1.24 mm^3^ for the high-dose group (Figure 1(b)). In these cases, the volume of GDNF distribution accounted for approximately 82% and 90% of the total striatal volume, respectively. Quantitative measures of the OD of GDNF staining confirmed the high levels of GDNF although no differences were noted between the low- and high-dose GDNF groups (Figure 1(d)).

**Figure 2:**
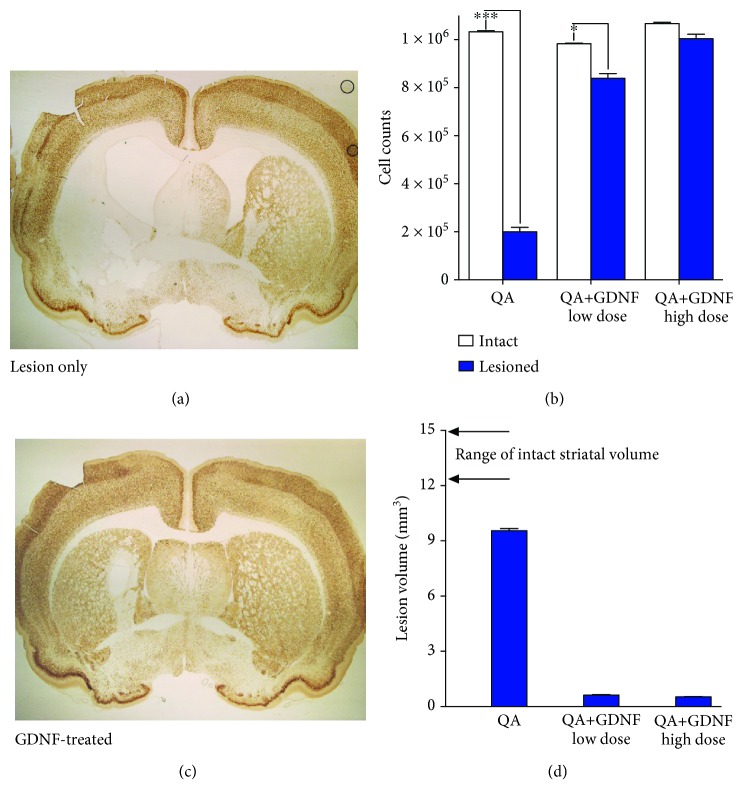
Protection of striatal neurons against QA toxicity. Photomicrographs of NeuN immunoreactivity illustrating an extensive loss of striatal neurons (a) that is largely prevented by prior implantation with the lower dose GDNF-secreting device (c). Quantification of striatal neurons (b) confirmed the extensive neuroprotection induced by GDNF with greater benefits observed in those animals receiving the higher dose implants. Quantification of the striatal volume further illustrated both the extensive nature of the QA lesion and the robust neuroprotection in GDNF-treated animals (d). ^∗^*p* < 0.05; ^∗∗∗^*p* < 0.001.

Immunohistochemical staining for NeuN confirmed that QA injections produced a marked spherical-shaped lesion that encompassed much of the striatum at the level of the injection (Figure 2(a)). Quantitative estimates of neuronal numbers confirmed both the magnitude of the QA lesion and the robust neuroprotection induced by GDNF. While neuronal numbers were decreased approximately 80% in QA-lesioned animals, this loss was largely prevented by GDNF with animals exhibiting a modest 15% and 6% neuronal loss in the low- and high-dose GDNF groups, respectively (Figure 2(b)). Lesion volume determinations showed a similar pattern with large volumetric losses induced by QA that were significantly prevented by GDNF (Figure 2(c)). QA injections produced a lesion that occupied approximately 65% of the total striatal volume. In contrast, treatment with GDNF resulted in a robust neuroprotective effect that manifests as small lesions encompassing only 4-5% of the implanted striatum (Figure 2(d)).

### 3.3. Behavioral Function

**Figure 3:**
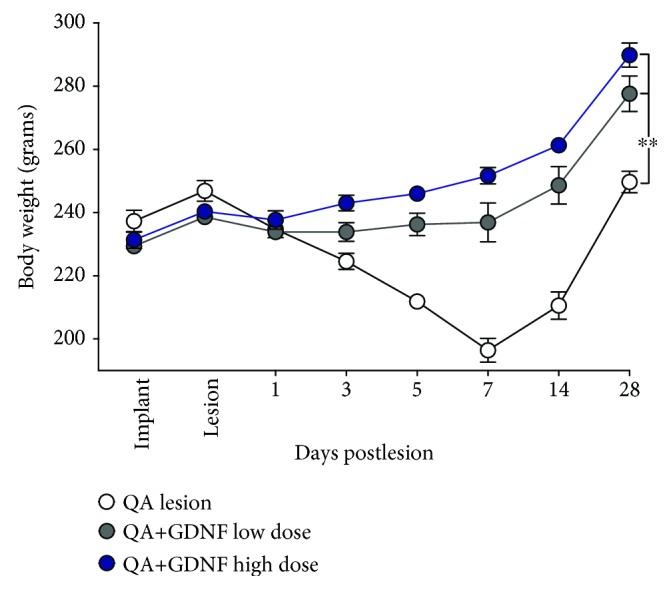
GDNF prevents the loss in body weight following QA lesion. QA alone produces a significant loss in body weight postlesion that is prevented by GDNF. While a trend towards greater benefit was observed with the higher secreting devices, this effect did not reach overall statistical significance. ^∗∗^*p* < 0.01.

QA produced a significant loss in body weight that was attenuated by treatment with GDNF (Figure 3). While nontreated animals lost >20% of their initial weight and did not begin to regain weight for approximately 1 week postlesion, the GDNF-treated animals exhibited only a transient loss of weight lasting for approximately 24 hours that was then followed by a typical pattern of weight gain thereafter. The loss in weight was attenuated by both doses of GDNF, and this benefit was slightly more pronounced, but not statistically significant, in the high-dose group.

**Figure 4:**
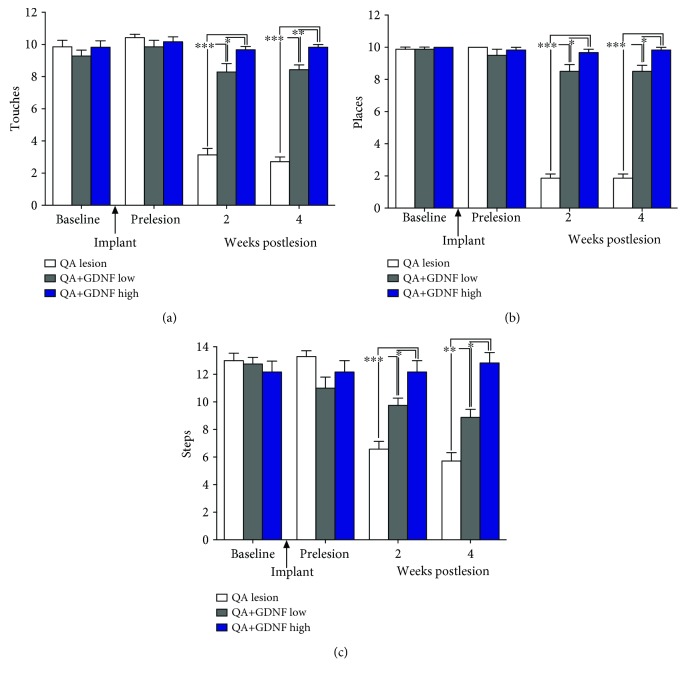
Performance in all the cylinder, placing, and stepping tests is significantly impaired at 2 and 4 weeks following intrastriatal injections of QA. In contrast, performance on each of these tests is preserved, in a dose-related manner, by EC-GNDF implants with the higher dose-treated animals performing comparably to presurgery levels (preimplantation and prelesion). ^∗^*p* < 0.05; ^∗∗^*p* < 0.01; ^∗∗∗^*p* < 0.001.

Tests of forelimb function in the cylinder, placing, and stepping tests confirmed that all groups of rats displayed normal forelimb use prior to the initiation of the study (preimplant; Figures 4(a)–4(c), respectively). Implantation of GDNF devices did not impact performance on any of the behavioral tasks. In contrast, the QA lesion produced significant behavioral deficits in all 3 tests. This effect was consistent and did not vary when the animals were tested at 2 versus 4 weeks postlesion. Relative to the intact forelimb, performance on the contralateral, impaired forelimb was decreased 69%-73%, 82%, and 49-56% (*p*′s < 0.05) in these control animals on the cylinder, placing, and stepping tests, respectively. In contrast, the GDNF-treated rats displayed a notable, dose-related improvement in performance with the higher dose of GDNF resulting in a virtually complete recovery of behavior.

## 4. Discussion

Every year millions of people worldwide are diagnosed with a neurodegenerative disorder that is ultimately fatal. Unfortunately, despite significant stepwise advances in our understanding of the underlying causes of many of these diseases, effective treatments have yet to be developed. This is particularly true for treatments capable of slowing or even reversing the insidious and progressive nature of many degenerative diseases. Despite identifying trophic proteins that protect and/or augment the function of targeted populations of neurons in multiple animal models of human diseases, the dream of translating preclinical success to the clinic has not yet been realized.

While many factors play a role in the difficulty of translating preclinical work into an effective therapy, an overriding issue across therapies is delivering proteins to the brain at therapeutic levels. Direct brain delivery is usually required due to the remarkably effective protective physiology and anatomy of the blood-brain barrier (BBB) that restricts entry of the majority of systemically delivered molecules [29]. Attempting to bypass this barrier by directly injecting drugs and proteins into the brain tissue also tends to be ineffective because poor diffusion from a point source of infusion reducing exposure of the targeted tissue and limiting any therapeutic impact [13]. These problems are compounded by the fact that systemically delivered drugs, in particular proteins, are large charged molecules that tend to be unstable and have a high propensity to aggregate and misfold that renders them ineffective and potentially toxic. Even if a protein can cross the BBB, it will be widely distributed and mistargeted throughout all brain parenchyma elevating the possibility for serious side effects such as those seen with mistargeting of proteins including the glial cell line-derived neurotrophic factor.

Here, we describe the use of polymer-encapsulated cells as a platform technology approach that has matured over the past 25 years and has now emerged as a viable therapeutic option capable of providing targeted, long-term, continuous, de novo synthesized delivery of very high levels of therapeutic molecules that can be distributed over significant portions of the brain [30]. In this approach, cells are enclosed or “encapsulated” within a capsule that has a semipermeable outer wall or membrane that can be implanted directly into the desired brain region [31–33]. The capsule wall morphology can be controlled to provide a pore structure that allows oxygen and nutrients to enter and nourish the cells while simultaneously providing a route for cell-secreted proteins, small molecules, antibodies, etc. to diffuse from the capsule and into the adjacent brain tissue. Immunological reactions that would typically occur against unencapsulated cells are prevented because the same porous structure that permits bidirectional flow eliminates entry of damaging elements of the host immune system into the capsule. Using human-derived cells even further eliminates any potential immunological reactions against the encapsulated cells. Of note, the present study used 2 different doses of GDNF obtained by simply extending the duration that the encapsulated cells were maintained in culture prior to implantation. We chose this pragmatic method only as a means of demonstrating a potential dose difference over the short duration of this study recognizing that such an approach would be insufficient for longer term dose differentiation as the encapsulated cells would simply reach capacity and equilibrate. Several more reliable and precise techniques of dose control could be used in clinical development including modifying the numbers of implants, changing the size of the devices including both the diameter and length, obliterating the center of the devices to alter the numbers of cells encapsulated, or selecting clonal cell lines with different secretion rates.

Intrastriatal injections of QA have been used as a model of Huntington's disease (HD) because the resulting excitotoxic lesion produces morphological changes similar to those seen in HD [34]. Trophic factors including GDNF and its family member neurturin have shown promise in animal models of several different neurodegenerative disorders, including HD [35–45]. The use of trophic factors has some unique appeal as a potential therapeutic in HD because genetic testing permits the identification of mutated gene carriers destined to suffer from HD [46]. Accordingly, identifying the genetic marker provides the potential opportunity to intercede prior to the development of symptoms secondary to neuronal degeneration. Still, the acute onset of toxicity produced by QA does not adequately capture the genetically driven onset and progression of neurodegeneration seen in the human disease, and the studies described here should be augmented with data obtained from studies using genetic mouse models of HD.

Ultimately, to be feasible, several essential prerequisites would need to be satisfied to treat the chronic and progressive nature of diseases such as HD. Here, we demonstrate that encapsulated cell technology can be used to provide widespread and targeted delivery of high levels of GDNF. These studies are enabled by the development of cell lines produced using a transposon-based gene expression system resulting in high GDNF secretion together with optimized cell scaffolding and membranes shown to promote long-term cell viability in vivo [21–23]. Additional studies have shown that the increases in striatal GDNF are persistent and stable for at least 6 months in the minipig putamen and 14 months in the rat striatum (the longest time point examined). The widespread GDNF diffusion was associated with pronounced behavioral protection and preservation of striatal anatomy as measured by sparing of NeuN-positive neurons and preservation of the striatal volume. While encouraging, subsequent studies should provide a more detailed analysis of striatal neuronal subtypes including more precise indices of individual neuron morphology and function. While not shown here, we have recently published several papers in animal models [31, 32], including excitotoxic lesions [33], that used GFAP immunohistochemistry to confirm the lack of inflammatory response following trophic factor delivery for longer periods of time than demonstrated in the current studies. Immunohistochemistry was also used to confirm GDNF receptor engagement. GDNF signals through a multicomponent receptor, first binding the GDNF family receptor*α*1 (GFR*α*1) with the resulting complex recruiting the transmembrane receptor kinase Ret or the neural cell adhesion molecule (NCAM) to initiate downstream signaling pathways. We found that treatment with GDNF dramatically increased the receptor expression and also highly increased RET phosphorylation [32].

Formal, GLP-compliant safety/toxicology studies conducted at Gloriana Therapeutics (unpublished data) have also confirmed the safety and tolerability of this approach by demonstrating that minipigs receiving bilateral implants of clinical-sized GDNF devices show no changes in food consumption/weight gain or behavior, no changes in blood chemistries, no production of anti-GDNF antibodies, and no surgery- or GDNF-related histopathological alterations were noted when the brains and peripheral organs were examined by a board-certified neuropathologist.

In conclusion, the encapsulated cell-based delivery system described here represents a greatly improved version of previous encapsulated cell-based systems to deliver trophic factors to the brain and to our knowledge is the first cellular delivery system capable of establishing the essential prerequisites of sustained, targeted, long-term delivery of sufficient quantities of GDNF to the CNS. As such, this approach represents a potentially novel and effective treatment for HD and other chronic neurodegenerative diseases.

## Data Availability

The experimental data used to support the findings of this study are included within the article.
